# Integrin and microtubule crosstalk in the regulation of cellular processes

**DOI:** 10.1007/s00018-018-2913-x

**Published:** 2018-09-11

**Authors:** Susan E. LaFlamme, Shomita Mathew-Steiner, Neetu Singh, Diane Colello-Borges, Bethsaida Nieves

**Affiliations:** 1 Department of Regenerative and Cancer Cell Biology, Albany Medical College, 47 New Scotland Avenue, Albany, NY 12208 USA; 2 Present Address: Indiana University, 975 W. Walnut Street, Indianapolis, IN 46202 USA

**Keywords:** Cell migration, Apical–basal polarity, Adhesion complexes, Focal adhesions

## Abstract

Integrins engage components of the extracellular matrix, and in collaboration with other receptors, regulate signaling cascades that impact cell behavior in part by modulating the cell’s cytoskeleton. Integrins have long been known to function together with the actin cytoskeleton to promote cell adhesion, migration, and invasion, and with the intermediate filament cytoskeleton to mediate the strong adhesion needed for the maintenance and integrity of epithelial tissues. Recent studies have shed light on the crosstalk between integrin and the microtubule cytoskeleton. Integrins promote microtubule nucleation, growth, and stabilization at the cell cortex, whereas microtubules regulate integrin activity and remodeling of adhesion sites. Integrin-dependent stabilization of microtubules at the cell cortex is critical to the establishment of apical–basal polarity required for the formation of epithelial tissues. During cell migration, integrin-dependent microtubule stabilization contributes to front–rear polarity, whereas microtubules promote the turnover of integrin-mediated adhesions. This review focuses on this interdependent relationship and its impact on cell behavior and function.

## Introduction

Integrins form a large family of α/β heterodimeric transmembrane receptors that bind to components of the extracellular matrix (ECM) such as fibronectin, collagen, and laminin [1–3]. Integrins function as both adhesion and signaling receptors allowing cells to respond to their ECM environment [1–3]. In collaboration with other receptors, integrins activate signal transduction cascades that regulate many cellular activities, including changes in gene expression and cytoskeletal assembly. In doing so, integrins regulate proliferation, survival, and migration at the cellular level, as well as morphogenesis, differentiation, and tissue homeostasis at the level of individual organisms [1–3].

Microtubules are cylinders formed from parallel protofilaments, each of which is composed of a polymer of α-tubulin and β-tubulin heterodimers [4, 5]. Microtubules can be nucleated at several subcellular sites [6–8] with the γ-tubulin ring complex (γ-TuRC) as the primary catalyst [7, 9, 10]. Microtubules are polarized filaments: their minus ends associate with centrosomes and spindle poles, and their plus ends exhibit dynamic growth and instability [4, 5], but can be stabilized by interactions with cellular structures including the cell cortex [4, 11]. There are many proteins that bind to and regulate microtubule growth, stability, and function; these are often referred to as microtubule-associated proteins or MAPs [4, 5].

The microtubule cytoskeleton is essential for many cellular processes [5]. Microtubules provide roadways for vesicular trafficking, as well as the positioning of intracellular organelles. They play essential roles in the establishment of front–rear polarity, which is necessary for directed cell migration [12], and contribute to the establishment of apical–basal polarity that is critical to the function of many different epithelial tissues [13, 14]. Microtubules are also the major structural components of the mitotic spindle, which together with microtubule motors mediate chromosome segregation at mitosis [15, 16]. This review will discuss the contribution of integrins to the mechanisms regulating microtubule nucleation at the centrosome and microtubule stabilization at the cell cortex both during cell migration and the establishment of apical–basal polarity, as well as the current understanding of how the assembly and turnover of integrin-mediated adhesions is regulated by changes in the microtubule cytoskeleton.

## Integrin signaling microtubule nucleation

Integrin-mediated adhesion initially impacts the microtubule cytoskeleton during the process of microtubule nucleation [17]. Microtubules can be nucleated from centrosomes and spindle poles, Golgi and nuclear membranes, as well as spindle microtubules, and chromatin [6, 7, 18]. The relative usage of these sites is regulated in a cell cycle- and cell type-dependent manner [8, 19, 20]. Microtubules are mostly nucleated by the γ-TuRC, which contains γ-tubulin and several associated proteins referred to as γ-tubulin complex proteins (GCPs). During nucleation, tubulin heterodimers bind to each other and γ-TuRC [7, 10, 21, 22]. Structurally, γ-TuRC positions γ-tubulin to nucleate the 13 protofilaments of a microtubule and caps the base (minus end) of each the newly polymerized microtubule [7, 10, 21, 22].

Studies from our laboratory identified a role for integrin signaling in the regulation of microtubule nucleation from interphase centrosomes. Cells adhered by a signaling-defective, mutant integrin exhibited defects in microtubule regrowth following nocodazole washout [23]. Later studies indicated that defects in microtubule regrowth were due to the suppression of microtubule nucleation [17]. Live-cell imaging demonstrated that the rate at which newly nucleated microtubules emanated from the centrosome was decreased in cells adhered by signaling-defective integrins [17]. Interestingly, the defect in microtubule nucleation correlated with a reduction in the recruitment of γ-tubulin to the centrosome, suggesting a mechanistic basis for the defect in microtubule nucleation.

Importantly, γ-tubulin is recruited to the centrosome as part of γ-TuRC. Thus, integrins may regulate the activation of specific signaling pathways that impact the assembly or recruitment and possibly the activity of γ-TuRC. Our signaling-defective mutant integrin inhibits the activation of the cytoplasmic tyrosine kinases FAK and Src, as well as the extracellular signal-regulated kinase, ERK [17, 24]. The roles of Src and ERK in regulating microtubule nucleation were further supported by inhibitor studies and molecular genetic approaches. Pharmacological inhibition of either Src or ERK for just 15 min resulted in rapid decline in γ-tubulin recruitment and microtubule nucleation when cells were adhered by wild-type integrins [17, 25], whereas molecular genetic approaches that resulted in the constitutive activation of Src or ERK restored the recruitment of γ-tubulin to the centrosome, as well as microtubule nucleation in cells adhered by mutant integrins [17, 25].

**Fig. 1:**
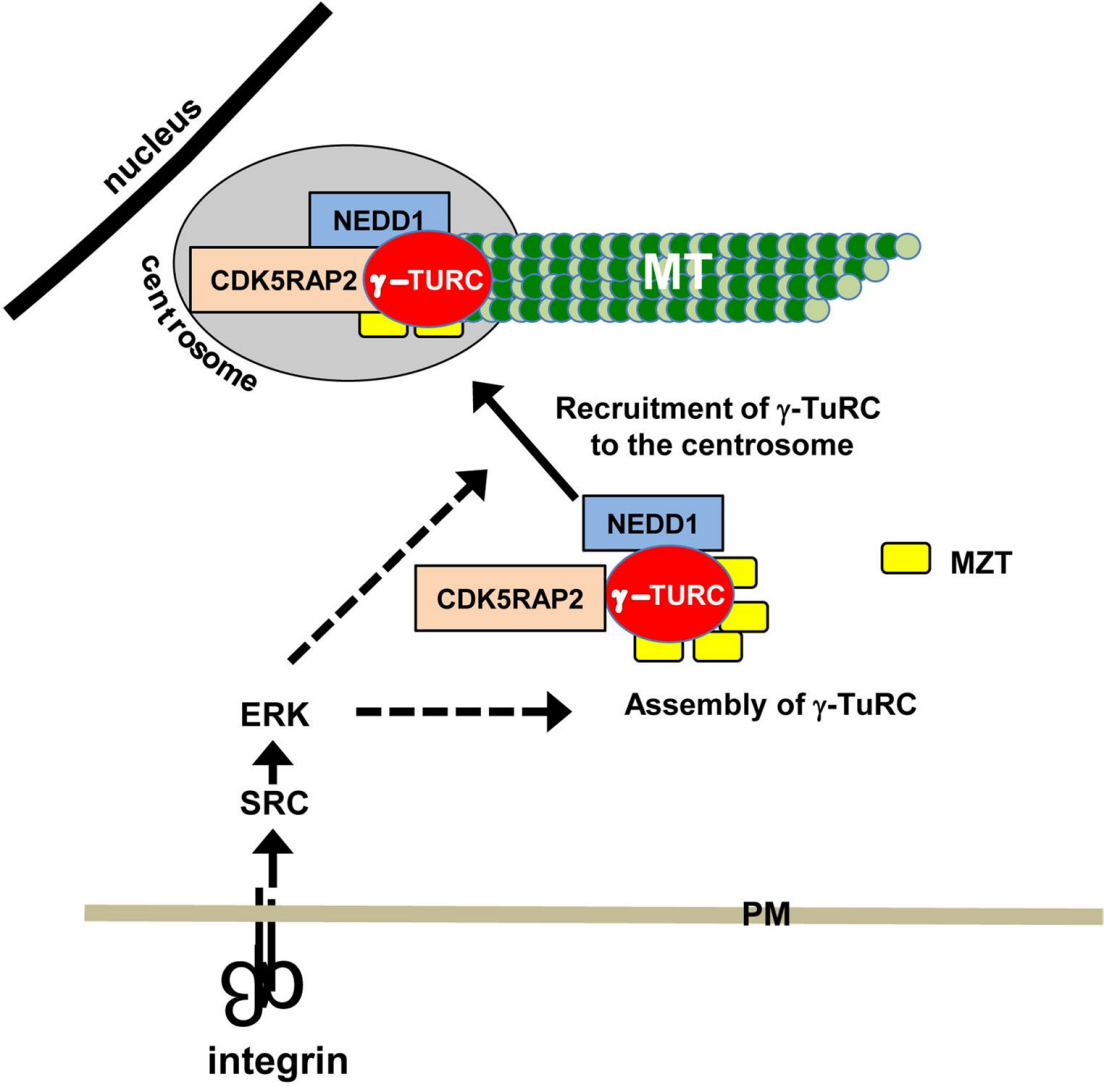
Regulation of microtubule nucleation by integrins. The integrin-dependent activation of SRC and ERK regulates microtubule (MT) nucleation at least in part by regulating the localization of γ-tubulin to the centrosome. γ-TuRC is assembled in the cytoplasm where it associates with additional proteins that regulate its centrosomal localization and microtubule nucleating ability. The integrin regulation of microtubule nucleation through SRC–ERK signaling could be due to integrin regulation of the assembly of the γ-TuRC complex, the association of this complex with NEDD1, CDK5RAP2 or MOZART (MZT) or the binding of the complex to the centrosome (dashed arrows). *PM* plasma membrane

The targets of integrin signaling that regulate γ-tubulin localization and microtubule nucleation have not yet been identified. It is important to note that γ-TuRC is assembled in the cytoplasm where it binds to several proteins that regulate its localization and nucleating activity. These include the following: NEDD1/GCP-WD) [26, 27], CDK5RAP2/CEP215 [28–30], and MOZART [31, 32]. The association of NEDD1/GCP-WD with γ-TuRC is required for the centrosomal recruitment of γ-TuRC and microtubule nucleation in many mammalian cell lines (Fig. 1) [26, 27]. The protein CDK5RAP2 activates the microtubule nucleating ability of γ-TuRC, presumably by inducing an “activated” conformation of γ-TuRC (Fig. 1) [10, 33–36]. MOZART (MZT) proteins associate with γ-TuRC and facilitate the interactions of NEDD1/GCP-WD and CDK5RAP2 with γ-TuRC [37, 38]. Thus, integrin signaling may impact γ-TuRC assembly, the binding of NEDD1/GCP-WD and MOZART, or the nucleating promoting activity of CDK5RAP2.

## Integrin-regulated microtubule growth and stability

Microtubule growth and stabilization are regulated by a complex network of proteins that bind at the plus end of microtubules often referred to as microtubule plus-end tracking proteins or +TIPS [4, 39]. Members of this group include the XMAP215 family of microtubule polymerases that promote microtubule growth by binding to plus ends and increasing the rate of tubulin addition by recruiting tubulin dimers [4, 39]. The end-binding (EB) proteins, including EB1 and EB3, are often used to track the plus end of growing microtubules. These proteins also serve as adaptors for the binding of other +TIPs, such as the cytoplasmic linker protein (CLIP)-associated proteins (CLASPs), and adenomatous polyposis coli (APC) [4, 39]. APC, EBs and CLASPs promote microtubule stabilization by linking the plus ends of microtubules to the cell cortex as discussed below.

The growth and stable interaction of microtubules with the cell cortex is a critical contributor to front–back cell polarity that is necessary for directed cell migration [12]. The family of small Rho-GTPases, Rho, Rac and Cdc42, are important regulators of microtubule growth and stabilization [40–46] and are activated downstream of integrin engagement [47–49]. Notably, integrin-dependent signaling is known to promote the stabilization of microtubules at the leading edge of migrating cells [45, 46, 50]. Depriving fibroblasts of integrin-mediated adhesion either by incubating them in suspension or by adhering them to a non-integrin ligand prevented the formation of stable microtubules [50]. Integrin-mediated adhesion activates FAK, and in fibroblasts, integrin–FAK signaling is required for microtubule stabilization, as FAK-null fibroblasts do not stabilize microtubules [50]. The Rho-dependent activation of the formin, mDia, contributes to the formation of a microtubule-stabilizing complex containing EB1, APC and IQGAP [41, 42, 44, 50], whereas integrin–FAK signaling restricts microtubule stabilization to the leading edge of migrating cells [50]. Interestingly, a similar link between integrins, mDia, IQGAP and microtubule stabilization occurs in mouse keratinocytes [51].

Signaling pathways can also promote microtubule stabilization by inhibiting the activity of proteins that destabilize microtubules. Stathmin is a microtubule-destabilizing protein [52]. The activity of stathmin is inhibited by phosphorylation at many sites by several different kinases, several of which are activated in an integrin-dependent manner, including p21-activated kinase (PAK), and ribosomal-S6 kinase-2 (RSK2) [52–54].

The glucogen synthase kinase 3β (GSK3β) is also a negative regulator of microtubule stabilization [12]. GSK3β phosphorylates several MAPs preventing their ability to bind to microtubules, including APC and CLASPs that stabilize microtubules at the cell cortex [55]. Importantly, the activity of GSK3β is suppressed at the leading edge of migrating cells, which allows these MAPs to bind and stabilize microtubules at the cell cortex [56]. Our studies showed that integrin-mediated adhesion of epithelial cells inhibits the activity of GSK3β through the activation of ribosomal S6 kinase RSK [60], whereas suppressing integrin activity leads to the inhibition of FAK and RSK, as well as a decrease in the number of EB1-positive microtubules at the cell surface [24, 57]. In fibroblasts, the Rho–mDia pathway inhibits GSK3β through the activation of a novel PKC [58]. Integrin activation of Cdc42 in astrocytes also leads to the inactivation of GSK3β, but does so via Par6 and the novel PKC, PKCζ [45, 46]. Thus, multiple pathways downstream of integrins in several cell types inhibit GSK3β to promote microtubule stabilization. Additionally, proteomic analysis of complexes associated with activated integrins indicates that these complexes are enriched for proteins associated with microtubule stabilization at the cell cortex including, EB1, IQGAP, mDia, CLASPs, and LL5α, (similar to LL5β, further supporting the role of integrins in microtubule stabilization [59]. The role of CLASPs and LL5β in integrin-regulated microtubule stabilization is discussed in more detail below.

## Integrins, microtubule stability, and apical–basal cell polarity

**Fig. 2:**
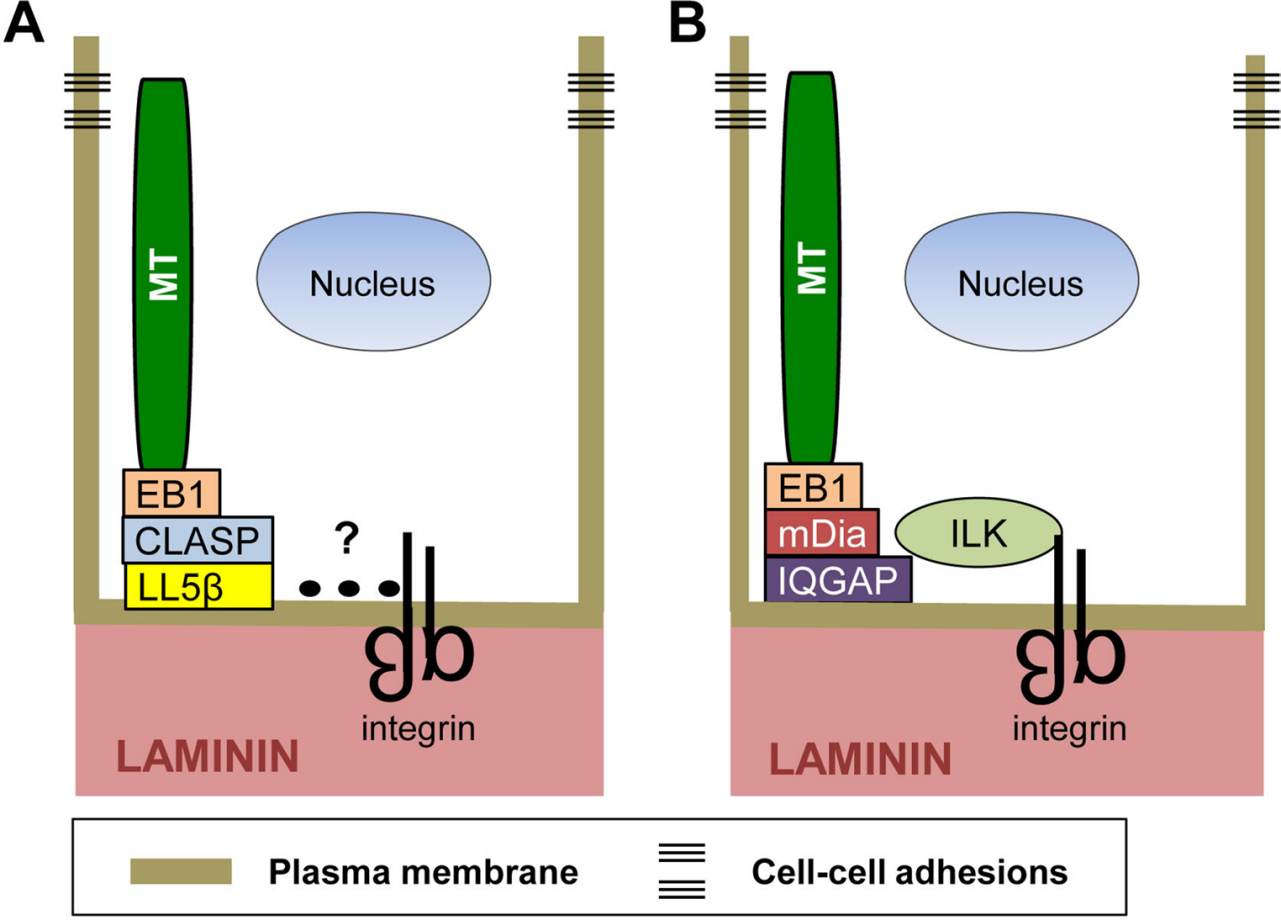
Integrins promote the stabilization of microtubules at the basal surface of polarized epithelial cells. Integrins stabilize microtubules at the basal cell cortex through two distinct protein complexes. **a** Microtubules bind to EB1, which binds to CLASP, which in turn binds to LL5β. LL5β is known to be present in a complex with α3β1 integrins; however, protein interactions (dotted line) mediating this association have not been identified. **b** Microtubules can also be associated with integrins through ILK, which is a cytoskeleton adaptor and integrin β subunit cytoplasmic domain-binding protein. ILK plays a role in stabilizing microtubules at the basal cell surface through a microtubule–EB1–mDia–IQGAP–ILK–integrin linkage

In epithelial tissues, noncentrosomal microtubules align along the apical–basal axis, with their plus ends captured and stabilized at the cell cortex apposed to the basement membrane [60]. Current evidence indicates that integrins play a necessary role in this process. Two recent studies employed mammary epithelial cells as a model to examine the mechanisms by which integrins establish the stable association of microtubule plus ends with the basal cell cortex [14, 61]. LL5β (and to a lesser extent LL5α), CLASP and EB1 were identified as key players in this process. In the human mammary epithelial cell line, MCF10A, as in other epithelial cells, integrins engage laminins in the basement membrane. LL5β and the laminin-binding integrin α3β1 colocalize at the basal cell cortex. Their basal localization is interdependent: inhibiting the expression of one suppresses the localization of the other [61]. Furthermore, integrin α3β1 and LL5β can be co-precipitated from cell lysates suggesting that they form a functional complex. The ability of α3β1 to form a complex with LL5β and to promote the localization LL5β to the basal cortex indicates that integrins stabilize the plus ends of microtubules at the basal cell cortex through a laminin–α3β1–LL5β–CLASP–EB1–microtubule linkage (Fig. 2a). However, there is no evidence to support the idea that α3β1 and LL5β directly interact with each other. It is possible that proteins involved in this linkage are similar to those found in migrating cells (discussed below).

Other studies have also demonstrated that integrins organize and stabilize the plus ends of microtubules at the basal surface of epithelial cells, which is necessary for epithelial polarity and lumen formation [13]. One study used primary luminal mammary epithelial cells (MECs) from transgenic mice, in which the integrin β1 gene was deleted [14]. Interestingly, MECs lacking β1 integrins are unable to form acini-containing lumens in 3D culture. In the absence of β1 integrins, EB1-labeled microtubules fail to localize to the basal cell surface. This is consistent with previous proteomic studies described above indicating that EB1 is present in complexes with activated integrins [59], as well as data from a proximal ligation assay demonstrating that β1 integrins and EB1 are closely associated at the basal surface [14]. Consistent with these findings, the inhibition of EB1 by RNAi technology also disrupts the establishment of epithelial polarity and the formation of lumens [14].

Many studies have shown that the cytoplasmic domain of the integrin β1 subunit can bind to cytoplasmic proteins to link integrins with the cell’s cytoskeleton and specific signaling pathways [62]. The integrin-linked kinase (ILK) is one such protein [63, 64]. Interestingly, the deletion of the ILK gene in MEFs also results in defects in the formation of acini in 3D culture similar to that which occurs when β1 integrins are deleted, suggesting that integrin–ILK linkages contribute to microtubule stabilization (Fig. 2b) [14]. The stabilization of microtubule plus ends at the basal cell surface promotes directed vesicular transport, which reinforces apical–basolateral polarity and promotes lumen formation [65]. Studies by others indicate that ILK can stabilize microtubules at the cell surface of keratinocytes by recruiting the scaffolding protein IQGAP and one of its effectors, mDia [51], previously shown to stabilize microtubules downstream of integrin engagement (Fig. 2b) [50].

## Microtubules regulate adhesion site assembly and turnover during cell migration

Focal adhesions are well-characterized sites where integrins engage components of the extracellular matrix to provide traction needed for cell movement and platforms for the assembly and activation of signaling complexes that regulate many aspects of cell behavior including cell migration [66, 67]. The formation of focal adhesions can be triggered by the depolymerimerization of microtubules, which activate tyrosine phosphorylation and the Rho GTPase, leading to enhanced integrin activation, the recruitment of specific adhesion complex components, and the assembly of focal adhesions [68–70].

The turnover or disassembly of focal adhesions to release integrin–matrix interactions is equally important for cell migration. Microtubules are critical to this process; their role was first described two decades ago when the repeated targeting of focal adhesions by microtubules was shown to trigger focal adhesion disassembly [71]. Interestingly, later studies showed that microtubule targeting of focal adhesions did not result in their disassembly in FAK-null fibroblasts, indicating that FAK plays a central role in focal adhesion turnover [72]. Dynamin and clathrin were also identified as key players, suggesting that the endocytosis of integrins may contribute to focal adhesion turnover [72, 73]. Interestingly, FAK binds to and recruits dynamin to focal adhesions; thus, FAK contributes to the positioning of the machinery necessary for the internalization of adhesion site components that accompanies adhesion site disassembly [72]. This is further supported by the finding that clathrin-dependent endocytosis of integrins coincides with focal adhesion disassembly with clathrin accumulating at focal adhesions during the initial stages of microtubule-induced focal adhesion turnover [73].

Microtubule stabilization at sites neighboring focal adhesions also contributes to adhesion site turnover. The disassembly of adhesion sites and the endocytosis of integrins require the disruption of the strong transmembrane linkage mediated by integrins between the actin cytoskeleton and the extracellular matrix. Current data suggest that the microtubule-dependent delivery and the subsequent secretion of matrix metalloproteases (MMPs) contribute to this process [74]. The localization of microtubule-associated CLASPs adjacent to focal adhesions has been temporally correlated with focal adhesion disassembly [74]. In migrating keratinocytes, a central role for CLASP–LL5β interactions has been identified in this process. LL5β (LL5α in some cells) is a cytoplasmic protein that associates with the cell cortex through the interaction of its Pleckstrin homology (PH) domain with the phospholipid, PI(3,4,5)P3 [75]. Importantly, LL5β can also bind CLASP, which in turn associates with EB1 bound to the plus end of growing microtubules. This association of LL5β, CLASP, and EB1 provides one mechanism linking the plus ends of microtubules to the cell cortex [76]. The role of these proteins in promoting focal adhesion turnover is further supported by the findings that the inhibition of CLASPs or LL5β using RNAi technology or the pharmacological inhibition of MMP activity prevents the turnover or focal adhesions in migrating keratinocytes [74].

**Fig. 3:**
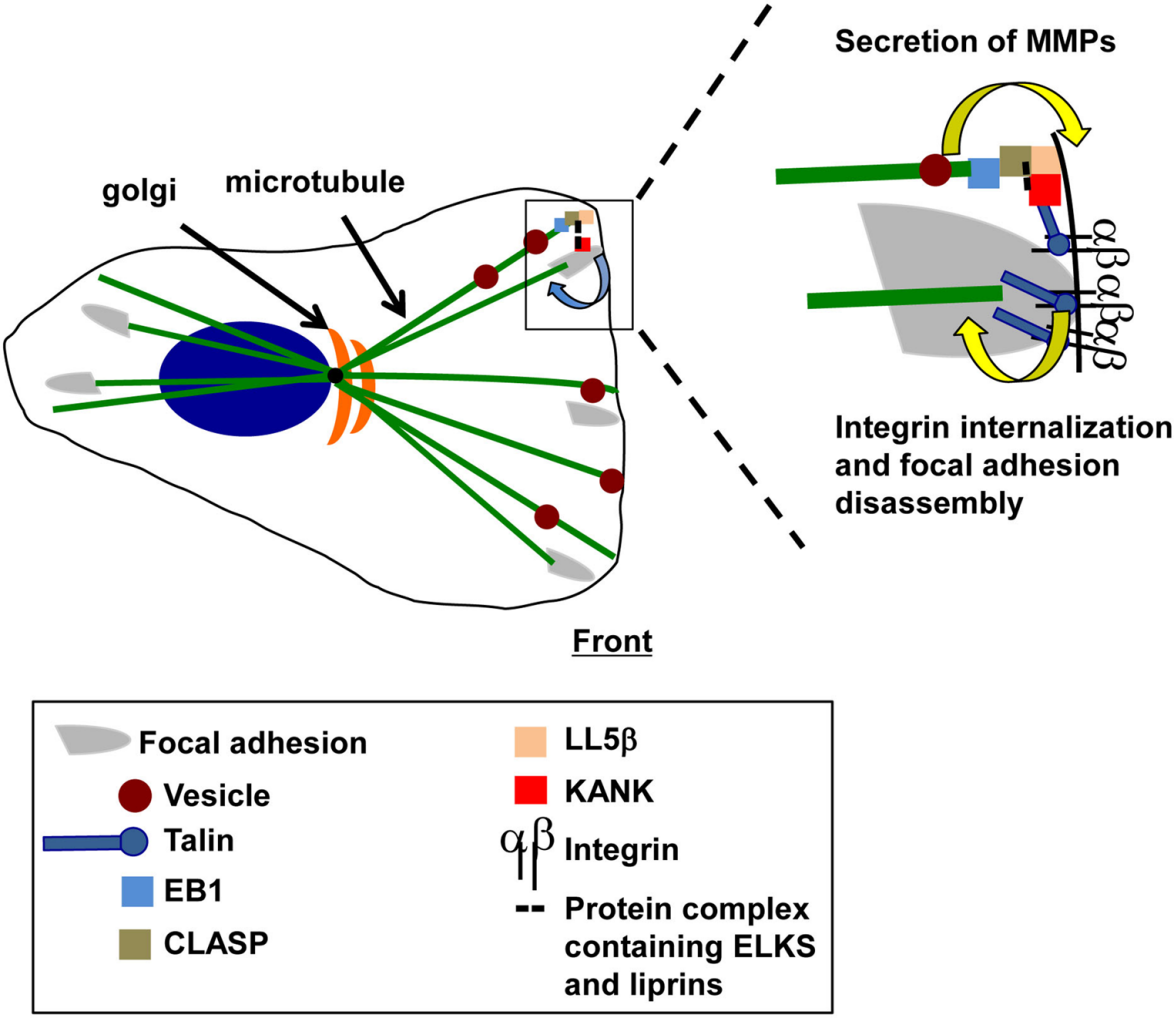
Microtubule–integrin crosstalk in migrating cells. Microtubules are stabilized at the rim of focal adhesions but are also thought to target focal adhesions directly to trigger adhesion site disassembly. Microtubules that are stabilized at the rim of focal adhesions deliver proteins including matrix metalloproteases (MMPs) for secretion to promote focal adhesion turnover. The proteins involved in this stabilization include EB1, CLASP, LL5β and a protein complex containing ELKS and liprins that link the plus ends of microtubules to KANK. Talin provides the linkage between KANK and integrins

The scaffolding proteins, liprins [77, 78], ELKs [79, 80] and KANKs [81], together with the kinesin family member, KIF21A [82], function together with EB1, CLASPs and LL5β to link microtubules to plasma membrane sites bordering focal adhesions (Fig. 3) [76, 82–84]. These sites have been referred to as cortical microtubule stabilization complexes (CMSCs) [84] and plasma membrane-associated platforms (PMAPs) [77, 83]. Interestingly, protein complexes that co-purify with activated integrin complexes contain microtubule-associated proteins, including EB1 and liprins, implicating activated integrins in the formation of CMSCs and PMAPs [59]. KANKs have been identified as critical players in the recruitment of CLASPs, LL5β and liprins to the rim of focal adhesions [84] and provide a mechanism linking CMSCs to integrins. Specifically, KANKs bind to talin, a cytoskeletal and focal adhesion protein that interacts with the β subunit cytoplasmic domains of integrins [1–3]. Thus, the KANK–talin–integrin association provides a mechanism to link microtubules and plasma membrane-associated complexes to integrin-mediated adhesions in migrating cells.

## Summary

The crosstalk between integrins and microtubules regulates cellular processes including directed cell migration, as well as the generation of epithelial apical–basal polarity and lumen formation during the morphogenesis of epithelial tissues. Integrins affect the microtubule cytoskeleton by promoting microtubule nucleation, growth, and stabilization, whereas microtubules impact integrin function by targeting integrin adhesions to promote their turnover. Although much has been learned about the mechanisms involved, interesting questions remain. For example, what are the protein targets of integrin signaling that promote the centrosomal recruitment of γ-tubulin and microtubule nucleation? NEDD1/GCP-WD, MOZART and CDK5RAP2 are intriguing candidates. Integrins promote the capture and stabilization of microtubules at cell cortex. Two distinct protein complexes have been characterized. Understanding whether these complexes function in the same cell or whether their assembly and function is context dependent is an important question that has yet to be addressed.

## Abbreviations

APC: Adenomatous polyposis coli
CDK5RAP2: Cyclin-dependent kinase 5-related activator protein 2
EB1: End-binding protein-1
ERK: Extracellular signal-regulated kinase
CLASP: Cytoplasmic linker protein (CLIP)-associated protein
FAK: Focal adhesion kinase
GCP: γ-tubulin complex protein
GSK-3β: Glucogen synthase kinase-3β
ILK: Integrin-linked kinase
IQGAP: IQ motif-containing GTPase-activating protein
KANK: KN motif and ankyrin-repeat-containing protein
KIF21A: Kinesin family member 21A
LL5β: Pleckstrin homology-like domain family B member 2
mDIA: Diaphanous-related formin-1
MMP: Matrix metalloproteinase
MOZART: Mitotic spindle organizing protein associated with a ring of γ-tubulin
NEDD1: Neural precursor cell expressed, developmentally down-regulated-protein 1
PAK: p21-activated kinase
PAR6: Partitioning-defective-6
PKC: Protein kinase C
RSK2: Ribosomal-S6 kinase-2
